# From Joint Loading to Osteoarthritis: A Multiscale Review of Knee Mechanobiology and Digital Modelling

**DOI:** 10.3390/ijms27167462

**Published:** 2026-08-20

**Authors:** Mikołaj Stańczak, Bartłomiej Kacprzak, Magdalena Hagner-Derengowska

**Affiliations:** 1 School of Mathematical and Computer Science, Heriot-Watt University, Edinburgh EH14 4AS, UK; 2 Sports Research Center, Nicolaus Copernicus University, 87-100 Toruń, Poland; 3 BK21 MedConcept, 90-349 Łódź, Poland; hipokrates@op.pl

**Keywords:** knee, mechanobiology, cartilage, mechanotransduction, PIEZO1, TRPV4, osteoarthritis, finite element modelling, machine learning, digital twin

## Abstract

The knee is a mechanically demanding synovial organ in which joint loading, tissue deformation, cellular mechanotransduction and matrix turnover are coupled. This narrative review critically links those scales and asks where the evidence is sufficiently mature for mechanistic or clinical inference. PubMed/MEDLINE and Europe PMC were searched from database inception to 20 July 2026 using structured terms for knee biomechanics, cartilage and osteochondral mechanobiology, finite element modelling, mechanosensitive channels, osteoarthritis, machine learning and digital twins. Landmark studies were selected for foundational models, while recent studies were prioritised for causal experiments, validation and translation. Instrumented implants show that common activities generate tibiofemoral forces of several times body weight, but tissue-level exposure also depends on muscle co-contraction, geometry and material properties. Biphasic and fibril-reinforced models explain how those loads become stress, strain, fluid pressure and osmotic signals. At the cell scale, TRPV4 and PIEZO1/2 participate in overlapping, context-dependent calcium signalling rather than a universal protective–pathological binary; most causal evidence remains preclinical. Osteoarthritis is therefore framed as a mechanically amplified feedback process involving cartilage, bone, synovium and systemic modifiers. Computational degeneration models and machine-learning surrogates are increasingly informative, although prospective validation, parameter identifiability and uncertainty propagation remain limiting. The review’s added value is an explicit transmission-and-validation framework that connects whole-joint observables to molecular responses while labelling the evidence source and translational readiness at every step.

## 1. Introduction

### 1.1. The Knee as a Multiscale Mechanobiological Organ

The knee is not a passive hinge. It is a mechanically and biologically integrated organ comprising articular cartilage, menisci, ligaments, subchondral and trabecular bone, synovium, synovial fluid and their resident cell populations [1,2,3,4]. During activity, whole-joint forces are redistributed by geometry and tissue architecture and appear locally as solid strain, fluid pressure, osmotic change, solute transport and interfacial shear. Cells interpret those signals and adjust matrix synthesis, degradation and remodelling, thereby changing how later loads are transmitted [5,6,7].

**Figure 1:**
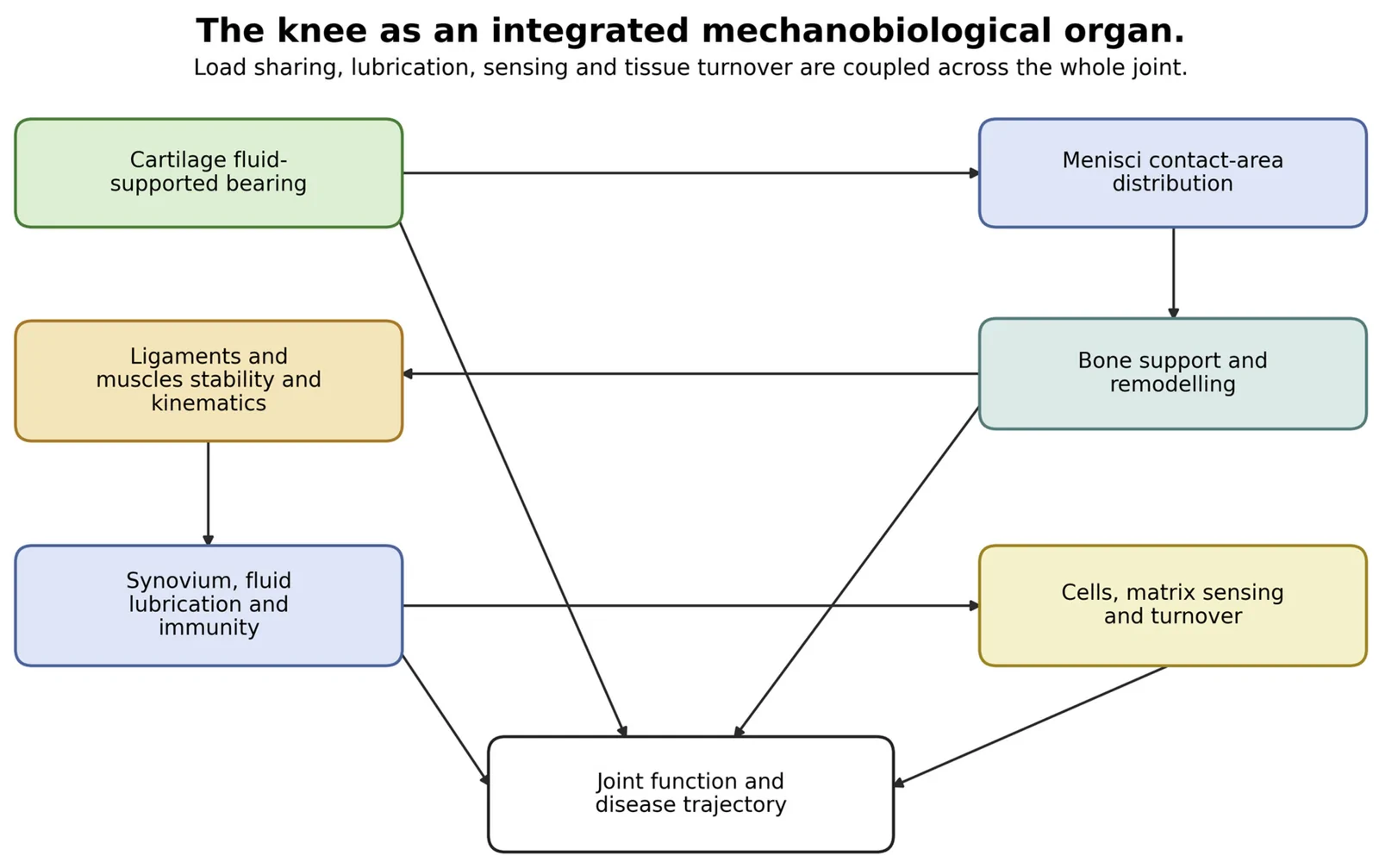
The knee as an integrated mechanobiological organ. Cartilage, menisci, ligaments and muscles, bone, synovium and resident cells jointly determine load sharing, lubrication, stability, tissue turnover and disease. Arrows indicate functional coupling rather than a single causal sequence.

Osteoarthritis (OA) is a failure of this coupled system rather than isolated cartilage wear. Injury, malalignment, obesity and ageing can alter joint mechanics; damaged matrix and synovial activation then modify cellular signalling, while changes in cartilage, meniscus and subchondral bone further distort the mechanical environment [4,8,9,10,11]. Figure 1 therefore treats the knee as an organ whose tissues have distinct functions but a shared disease trajectory.

### 1.2. Conceptual Advance, Audience and Scope

Existing reviews usually emphasise one domain: joint kinematics and loading, cartilage constitutive mechanics, chondrocyte mechanotransduction, OA biology or computational modelling. The present review adds a different organising device: a transmission-and-validation framework. It follows the quantity that passes between scales from motion and force, to stress and fluid pressure, to membrane and cytoskeletal signals, to gene regulation and identifies the experimental observation that can validate each transfer. This makes assumptions and uncertainty visible instead of treating separate studies as if they formed a validated continuous chain.

In brief, the review concludes that whole-joint loads are well measured but derive from a small arthroplasty population, that tissue- and cell-level mechanisms are increasingly well characterised yet remain largely preclinical, and that current machine-learning and digital-twin tools are promising rather than clinically validated. Progress therefore depends less on adding further mechanisms than on measuring the links between scales and quantifying their uncertainty.

The review is intended for molecular and cellular scientists who need the mechanical provenance of an experimental stimulus, and for biomechanical modellers who need to know which biological responses are causal, associative or still hypothetical. Only three scale-bridging equations are retained in the main text. More detailed formulations, assumptions and validity domains have been moved to Appendix A. The scope remains broad, but every topic is included only insofar as it explains load transmission, mechanosensing, OA feedback or the validation of predictive models.

### 1.3. Review Strategy

This article is a critical narrative review. PubMed/MEDLINE and Europe PMC were searched from database inception to 20 July 2026. Search blocks combined knee, tibiofemoral, patellofemoral or osteoarthritis with biomechanics, kinematics, contact force, musculoskeletal model, finite element, biphasic, triphasic, cartilage, meniscus, ligament, subchondral bone, synovium, mechanotransduction, calcium, primary cilium, integrin, YAP/TAZ, TRPV4, PIEZO1, PIEZO2, RANKL, sclerostin, machine learning or digital twin. Reference lists of foundational and recent papers were examined to identify antecedent models and validation studies.

Peer-reviewed human studies, human tissue studies, animal experiments, in vitro or ex vivo experiments and computational studies were eligible when they addressed the load-to-biology chain in the knee. Non-knee studies were retained only when they established a constitutive theory or a general mechanotransduction mechanism used directly in knee research. Conference abstracts, claims without an extractable method or outcome, and studies without direct mechanobiological relevance were excluded. Landmark studies were selected for foundational theory or first causal demonstration; publications from 2020 to 2026 were prioritised for updated mechanisms, therapeutic translation, machine learning and digital twins. Because no quantitative meta-analysis or formal risk-of-bias scoring was performed, completeness should not be inferred as for a systematic review.

Evidence is labelled throughout as human clinical, human tissue/ex vivo, animal in vivo, in vitro, or computational. Causal language is reserved for perturbation experiments or interventions within the model studied. Associations in human tissue, successful prediction of a simulated endpoint and protection in a mouse OA model are not treated as equivalent evidence for a human disease-modifying therapy.

## 2. Whole-Joint Kinematics and Kinetics

### 2.1. Kinematic Conventions and Joint Coordinate Systems

The tibiofemoral joint permits motion in six degrees of freedom: three rotations—flexion–extension, internal–external rotation and abduction–adduction (varus–valgus)—and three translations—anterior–posterior, medial–lateral and proximal–distal [12]. The patellofemoral joint adds a second articulation through which the quadriceps transmits its force to the tibia via the patellar tendon, the patella acting as a sesamoid that increases the moment arm of the extensor mechanism. During flexion the femoral condyles simultaneously roll and slide upon the tibial plateau, with the lateral condyle translating posteriorly more than the medial, producing the coupled axial rotation that characterises natural knee motion [13,14,15].

Terminal extension is accompanied by an obligatory external rotation of the tibia relative to the femur, the screw-home mechanism, which brings the joint into a close-packed, stable configuration [16]. Because the instantaneous axis about which the joint rotates is not fixed but migrates throughout the range of motion, the kinematics are most rigorously described by the path of the instantaneous helical, or screw, axis [17]. Standardised joint-coordinate recommendations, anatomy-based coordinate systems, passive-motion studies and loaded fluoroscopic or magnetic-resonance measurements provide complementary descriptions [18,19,20,21,22].

The Grood–Suntay joint coordinate system defines a femoral flexion axis, a tibial axial-rotation axis and their common perpendicular, or floating axis [23,24]. Its clinically interpretable rotations and translations remain the field standard, although extreme flexion and hyperextension require corrected formulations [25]. The mathematical definition is provided in Appendix A rather than interrupting the biological narrative. A crucial limitation is that coordinate-system choice, landmark definition, skin-motion artefact and loading condition can all change the reported kinematics; values are therefore not interchangeable across protocols [13,14,15,16,17,18,19,20,21,22].

### 2.2. Inverse Dynamics and Equations of Motion

Inverse dynamics combines segment kinematics, external forces and body-segment inertial parameters to estimate net intersegmental forces and moments [26,27,28,29,30]. For a multibody system with generalised coordinates q, the essential relation is

(1) *M(q)q̈* + *C(q,q̇)q̇* + *G(q)* = *τ*

where M is the mass matrix, C contains velocity-dependent terms, G contains gravity and τ is the vector of net generalised loads [31]. Equation (1) assumes a chosen rigid-body representation and does not identify individual muscle, ligament or articular-contact forces. Its biological relevance is therefore indirect: it provides the external and net-joint constraints from which tissue loading is inferred. Errors in motion tracking, inertial parameters and ground-reaction forces propagate into all downstream estimates.

### 2.3. In Vivo Knee Loads

Instrumented total knee replacements (TKRs) provide the most direct in vivo measurement of tibiofemoral contact force in replaced rather than native knees, and remain the principal validation anchor for musculoskeletal models [32,33,34,35]. In the canonical five-subject dataset, mean peak resultant force ranged from approximately 107% of body weight (BW) during two-leg standing to 346% BW during stair descent (Table 1) [33]. Muscle co-contraction explains why internal contact force substantially exceeds the force inferred from the external knee moment alone [34,36].

These measurements should not be treated as universal native-knee norms. They derive from small cohorts with arthroplasty, altered articular geometry and individual motor strategies. Speed, implant alignment, pain, muscle co-contraction and body size contribute to between-subject variability [32,33,34,35,36,37,38,39,40,41,42,43,44,45]. The external knee adduction moment is useful at group level but is an imperfect proxy for medial contact force because it omits lateral load and much of the muscular contribution [36,45,46,47].

### 2.4. Musculoskeletal Modelling and Muscle Redundancy

More muscles cross the knee than there are mechanical degrees of freedom, so individual muscle forces cannot be recovered from net moments without additional assumptions [48,49]. Static or dynamic optimisation typically minimises an activation- or stress-based cost subject to moment-balance and physiological constraints [50]. Electromyography (EMG)-driven and EMG-assisted approaches replace part of the optimality assumption with measured excitation, improving representation of co-contraction but adding calibration and measurement uncertainty [46,47].

Hill-type musculotendon actuators combine activation, force–length, force–velocity, passive elasticity and pennation [31,51,52,53]. They are embedded in platforms such as OpenSim and the AnyBody Modeling System and in validated lower-limb or whole-body models [53,54,55,56,57,58,59,60,61,62,63,64,65]. The detailed optimisation and Hill relations are listed in Appendix A. For mechanobiology, the important output is not the actuator equation itself but the subject- and task-specific muscle force that becomes a boundary condition for joint-contact and tissue-scale models.

### 2.5. Joint Contact Mechanics

Classical Hertz theory provides an instructive instantaneous estimate of contact area and peak pressure for curved elastic bodies [66,67], but its assumptions small strain, smooth non-conforming surfaces, linear elasticity and no interstitial fluid do not describe articular cartilage over physiological time scales. Biphasic contact theory instead predicts high initial fluid support and gradual transfer of load to the solid matrix [68]. Elastic-foundation models offer speed for musculoskeletal coupling, whereas finite element (FE) contact models resolve heterogeneous cartilage and meniscal deformation at substantially greater computational cost [69,70,71,72,73,74,75,76,77,78,79,80,81,82,83].

The choice of contact formulation is biologically consequential. A model that reproduces total joint force can still misestimate local stress, fluid pressure or fibril strain and therefore supply the wrong stimulus to a mechanotransduction model. Validation must match the intended output: implant telemetry tests resultant force, pressure sensors or imaging test regional contact, and tissue experiments test constitutive response.

## 3. Tissue-Scale Mechanics and Constitutive Modelling

Joint forces become spatial fields of stress, strain, fluid pressure and solute transport through tissue-specific material laws. Cartilage and meniscus are hydrated fibre-reinforced composites; ligaments are anisotropic and viscoelastic; bone remodels; and the synovial interface couples fluid rheology to boundary lubrication. Table 2 compares the principal model classes and makes explicit what each can and cannot represent.

### 3.1. Articular Cartilage: Biphasic Theory

The foundational biphasic theory represents cartilage as an intrinsically incompressible porous solid matrix saturated with incompressible interstitial fluid [84]. Time-dependent creep and stress relaxation then arise largely from frictional drag as fluid moves through the matrix, not solely from intrinsic viscoelasticity. The scale-bridging relation retained here is Darcy’s law,

(2) *q* = −*k∇p*

where q is relative fluid flux, k is hydraulic permeability and *p* is pore pressure [84,85,86]. Equation (2) assumes continuum-scale flow, a specified permeability law and negligible inertia. It is valid at the tissue scale, not at an individual molecular pore. Biologically, it explains how cyclic loading produces fluid pressurisation, convection and time-dependent cell exposure. The characteristic consolidation time increases with specimen thickness squared and decreases with the product of aggregate modulus and permeability; geometry and testing protocol therefore contribute substantially to reported variability [87,88,89,90,91,92,93,94,95,96,97,98,99,100,101].

The biphasic theory was validated against confined and unconfined compression and against indentation, the last requiring a numerical solution that established how the instantaneous response is governed by the solid shear modulus while the equilibrium response is governed by the aggregate modulus [87,88,89]. A key corollary, since confirmed experimentally, is that the interstitial fluid carries the overwhelming majority of the applied load immediately after loading, shielding the solid matrix and the cells within it from the full stress and accounting for cartilage’s remarkable durability and low friction [91].

The three canonical testing configurations confined compression, unconfined compression and indentation probe complementary aspects of this behaviour and together allow the full set of material parameters to be identified. In confined compression, the tissue is laterally constrained in a rigid chamber and loaded through a porous platen, so fluid can escape only along the loading axis. The response is then governed by the aggregate modulus and the permeability, which yields the cleanest measurement of the consolidation behaviour [84]. In unconfined compression between impermeable platens, fluid escapes radially. Lateral expansion engages the collagen network in tension, making the response sensitive to tension–compression nonlinearity and fibril reinforcement.

This configuration exposed the limitations of isotropic theory [89]. Indentation can be performed on intact joint surfaces, but the response depends on local thickness, modulus, permeability and indenter geometry. Its analysis required a numerical biphasic solution, establishing the method as a means of characterising cartilage in situ [87,88]. The convergence of these independent configurations on a consistent set of properties is one of the strengths of the biphasic framework and the basis for confidence in the parameters used in whole-joint models [91].

### 3.2. Charged and Fibril-Reinforced Cartilage Models

Two limitations of the linear biphasic theory motivated its principal extensions. The first is that cartilage is electrically charged: the proteoglycans carry a dense fixed negative charge that, by drawing in mobile counter-ions, generates a Donnan osmotic swelling pressure that pre-stresses the tissue and contributes to its compressive stiffness. Lai, Hou and Mow incorporated this physics in the triphasic theory [102]. It adds an ion phase, comprising the cation and anion of a neutral salt, to the solid and fluid phases. Total stress is partitioned among solid-matrix elasticity, Donnan osmotic pressure and chemical-expansion stress.

Fixed charge density, of order two to three tenths of a milliequivalent per millilitre, sets the osmotic contribution. The theory thereby explains cartilage swelling, stiffness dependence on bathing-solution concentration and electrokinetic streaming potentials generated by load-induced fluid flow [102,103]. Ateshian and colleagues established the formal correspondence between the equilibrium moduli of the biphasic and triphasic descriptions, clarifying how the osmotic and elastic contributions combine [104]. Multiphasic generalisations further separate multiple ionic species and are reviewed comprehensively in the cartilage modelling literature [105].

The second limitation is that the linear theory treats the solid as isotropic, whereas cartilage is strongly anisotropic and far stiffer in tension than in compression, owing to its depth-varying collagen architecture. Fibril-reinforced poroelastic and poroviscoelastic models address this by superimposing on the biphasic matrix a network of collagen fibrils, represented either as discrete springs or as a continuous angular distribution, that bear tensile but not compressive load [106,107].

In these models the collagen network governs the dynamic and tensile response and the apparent Poisson effect, while the proteoglycan-rich ground substance governs the equilibrium compressive response. The tension–compression nonlinearity therefore emerges naturally, and the contributions of the two principal constituents can be separated and identified from experiment [107]. Conewise linear elasticity provides a formal continuum framework for this bimodular behaviour [105]. Fibril-reinforced poroviscoelastic models add intrinsic collagen viscoelasticity to flow-dependent dissipation. They have characterised healthy and osteoarthritic human cartilage and revealed the collagen-network degradation and proteoglycan loss that accompany disease [99].

Related nonhomogeneous fibril-reinforced formulations and analyses of the role of interstitial fluid pressurisation complete this picture of the tissue as a fibre-reinforced, fluid-pressurised composite [96,108].

No single formulation is uniformly superior. Triphasic models are justified when osmotic swelling, ion transport or fixed charge is central; fibril-reinforced models are needed for collagen strain, tensile damage or depth-dependent anisotropy. Adding mechanisms without independent data can worsen identifiability, so calibration against only one compression curve is insufficient for a highly parameterised model [92,93,94,95,96,97,98,99,100,101,103,104,105,106,107,108,109].

### 3.3. Meniscus

The menisci are wedge-shaped fibrocartilages whose function in load transmission depends on a highly organised collagen architecture: coarse circumferential bundles of type I collagen, anchored at the anterior and posterior root insertions, are reinforced by radial tie fibres [110]. Under axial compression the wedge is squeezed and tends to extrude radially, but the circumferential fibres resist this extrusion and develop tensile hoop stress, converting compression into circumferential tension and thereby distributing load over a large area of cartilage.

The circumferential tensile modulus is correspondingly high, of order one hundred to three hundred megapascals, while the radial and transverse moduli are far lower, a pronounced anisotropy that is captured by transversely isotropic and fibre-reinforced poroelastic constitutive models [110,111]. The mechanical consequence of this architecture is that lesions which sever the circumferential fibres, such as radial tears and root avulsions, abolish hoop-stress transmission and sharply elevate cartilage contact stress, whereas the tissue can tolerate considerable damage that spares the load-bearing fibres before contact mechanics deteriorate.

The loss of meniscal function, whether through degeneration, tearing or surgical resection, is among the most powerful mechanical drivers of knee osteoarthritis. Meniscal structure, composition and function have been studied extensively in experiment and finite element simulation. This literature covers contact pressure, anisotropic constitutive models and the mechanical consequences of tears and meniscectomy [112,113,114,115,116,117,118,119,120,121,122,123].

### 3.4. Ligaments and Tendons

Knee ligaments and tendons are nonlinear, anisotropic and viscoelastic. Their low-stiffness toe region reflects collagen crimp recruitment, followed by a stiffer approximately linear response [124,125]. Quasi-linear viscoelasticity (QLV) separates an instantaneous nonlinear response from a reduced relaxation function, whereas anisotropic hyperelastic models derive stress from a strain-energy function with one or more collagen-fibre families [126,127,128,129,130,131,132,133,134,135,136,137].

The Holzapfel–Gasser–Ogden family can represent fibre dispersion and tension-only recruitment, but a form developed for arterial wall should not be transferred to a ligament without redefining the reference configuration, fibre architecture and loading data. The corrected QLV and HGO-type expressions are given in Appendix A. Whole-knee predictions remain sensitive to insertion sites, pre-strain and slack length as well as to material constants [82,128,129,130,131,132,133,134,135,136,137].

### 3.5. Subchondral Bone and Mechanically Regulated Remodelling

Subchondral and trabecular bone are living, poroelastic and continually remodelling tissues whose architecture adapts to mechanical demand. Changes in OA including subchondral sclerosis, accelerated turnover and altered trabecular structure are integral to disease rather than incidental to it [138]. Apparent trabecular-bone stiffness scales steeply with apparent density, classically through a Carter–Hayes-type power law [139]. Frost’s mechanostat describes adaptation through tissue-dependent strain windows: disuse favours resorption, an intermediate range maintains mass, higher strain favours formation, and near-fracture exposure permits damage to outpace repair [140,141]. These boundaries are phenomenological rather than universal molecular thresholds.

Huiskes, Weinans and colleagues gave this concept computational form by relating local change in apparent density to the mismatch between strain-energy density per unit mass and a homeostatic target, with a lazy zone in which no net remodelling occurs [142,143]. The equation and its validity assumptions are given in Appendix A. Such laws can reproduce trabecular density patterns and predict stress shielding around stiff implants, but they aggregate osteocyte sensing and multicellular remodelling into fitted parameters. Bone adaptation is therefore a clear mechanobiological feedback example, not a direct molecular readout [142,143].

At the cellular level, these phenomenological laws summarise the behaviour of the osteocyte network. Osteocytes are embedded in the mineralised matrix and interconnected by dendritic processes within the lacunocanalicular system, a geometry well suited to detecting the interstitial fluid flow generated by loading [2]. Load-induced fluid shear and matrix strain are transduced at the cell membrane, primary cilium and dendritic processes, and are relayed between cells through connexin-based gap junctions and hemichannels, allowing the network to integrate a mechanical signal across the tissue [2]. Activated osteocytes then adjust the sclerostin, RANKL and osteoprotegerin signalling that couples local strain to osteoblast-driven formation and osteoclast-driven resorption, as detailed in Section 4.3 [2,144]. This lacunocanalicular pathway is the biological substrate of the strain windows and lazy zones that the remodelling equations above represent only phenomenologically.

The mechanical coupling between cartilage and the bone beneath it adds a further dimension to this loop that is central to osteoarthritis. The subchondral plate and trabecular bone are far stiffer than cartilage, so their properties influence stress in the overlying tissue. Sclerosis in established disease may reduce load attenuation and increase cartilage stress. Early disease can instead involve accelerated remodelling and relative softening [138].

Computational models of the osteochondral unit that represent both tissues and their interface predict how changes in subchondral bone properties redistribute stress in the cartilage. They suggest that the bone and cartilage changes in osteoarthritis are mechanically interdependent rather than independent consequences of a common insult [145]. This interdependence is the structural counterpart of the biological bone–cartilage crosstalk described later in this review, and it illustrates why a model of the knee that represents cartilage in isolation, with a rigid foundation, may misrepresent the very stress fields that drive the disease [146].

Mechanostat thresholds and strain-energy-density adaptation laws are useful phenomenological descriptions, not invariant biological constants. They compress osteocyte sensing, osteoblast–osteoclast coupling, vascular supply and endocrine regulation into a small number of parameters [138,139,140,141,142,143,145,146,147,148,149,150,151,152,153,154]. Their predictions should therefore be interpreted as hypotheses about spatial adaptation rather than direct molecular readouts.

### 3.6. Synovial Fluid and Cartilage Lubrication

The exceptionally low friction of healthy articular cartilage with coefficients well below those of engineered bearings is achieved by a combination of mechanisms that depend on both the synovial fluid and the biphasic nature of the tissue. Synovial fluid is a non-Newtonian, shear-thinning, viscoelastic fluid whose rheology derives chiefly from high-molecular-weight hyaluronic acid and from the mucinous glycoprotein lubricin, the product of the PRG4 gene, which together provide boundary lubrication at the cartilage surface [155,156]. The Stribeck framework relates friction to viscosity, sliding speed and load [86]. It distinguishes boundary lubrication, in which surface-bound molecules carry more load, from low-friction fluid-film lubrication, with a mixed regime between.

For soft, permeable cartilage the classical Stribeck picture is modified into an elastoviscous transition in which lubricin and hyaluronic acid act synergistically, lubricin concentrating hyaluronic acid at the surface and providing boundary protection [157]. Biphasic, or interstitial-fluid-pressurisation, lubrication acts in parallel with these surface mechanisms. Pressurised cartilage fluid carries most of the contact load, leaving only a small fraction for direct solid contact and maintaining low effective friction during cyclic activity [158]. The breakdown of these lubrication mechanisms, through loss of lubricin or hyaluronic acid or through changes in cartilage permeability, contributes to the surface wear of early osteoarthritis.

The mechanisms of joint lubrication, the roles of its molecular constituents, and the modes of friction and their experimental verification have been characterised in detail [71,159,160,161,162,163,164].

### 3.7. Whole-Knee Finite Element Models and Parameter Variability

The constitutive theories of the preceding subsections are the material laws required by finite element models, which assemble them into subject-specific geometries reconstructed from magnetic-resonance or computed-tomography imaging to compute the detailed stress, strain, fluid-pressure and contact fields throughout the joint [70]. The reproducibility and validation of such models have been longstanding concerns, addressed in part by the Open Knee(s) project, which provides a freely available library of specimen-specific knee finite element models and the associated experimental data for validation [72,73]. Current pipelines couple musculoskeletal and tissue-scale models.

Muscle and contact forces estimated from gait can drive a finite element model with fibril-reinforced poroelastic cartilage and menisci. The output is a distribution of stress, fibril strain and interstitial fluid pressure across stance [74]. Such combined pipelines are the computational embodiment of the cross-scale loop, carrying organ-scale loads down to the tissue-scale fields that, as the next sections describe, the cells of the joint actually experience. A wide range of subject-specific and explicit finite element knee models, differing in their representation of ligaments, menisci and contact and in their validation against experiment, has been developed for both natural and replaced knees [75,76,77,78,79,81,82,83].

Finite element credibility is output specific. Geometry may be checked against imaging, ligament response against laxity tests, resultant force against telemetry and contact area or pressure against cadaveric or imaging measurements. A model validated for global kinematics is not automatically validated for chondrocyte-level fluid pressure. Open Knee(s) and related benchmark data improve reproducibility, but differences in segmentation, mesh, contact, boundary conditions and parameter mapping still produce substantial dispersion [70,71,72,73,74,75,76,77,78,79,80,81,82,83].

**Figure 2:**
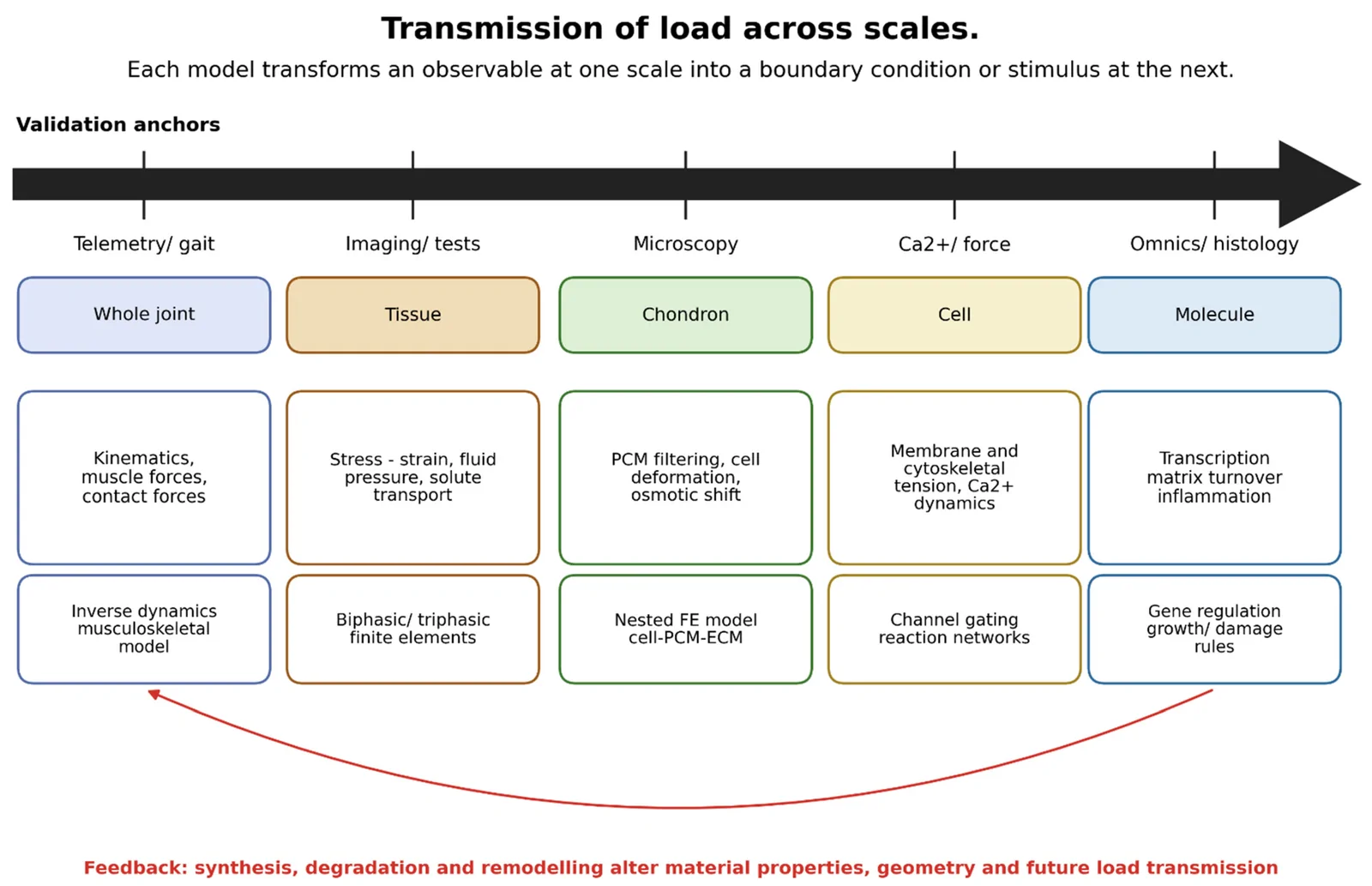
Transmission of load across scales. Whole-joint models estimate forces; constitutive and finite element models transform them into tissue fields; nested models estimate chondron and cell stimuli; and signalling models relate those stimuli to matrix turnover. The upper strip lists output-specific validation anchors. Feedback from turnover to material properties closes the loop.

Table 3 reports representative ranges to show the scale of this uncertainty, not to define universal healthy values. Figure 2 summarises the cross-scale transfer and the distinct validation anchor required at each stage.

## 4. Cell Biology of Knee Tissues

Mechanical fields matter because cells build, sense and degrade the joint. The principal populations are chondrocytes in cartilage; osteocytes, osteoblasts and osteoclasts in bone; fibroblast-like and macrophage-like synoviocytes; and fibroblasts or progenitors in ligaments, entheses, marrow and synovium [1,2,3,5,144,178,179,180,181,182,183,184,185,186,187,188,189,190,191,192,193,194,195,196,197,198,199,200,201,202,203,204,205,206,207,208,209,210,211,212,213,214,215,216,217,218,219,220,221,222,223,224,225,226,227,228,229,230,231,232,233,234,235,236]. Their responses are tissue-specific and are modified by metabolic and inflammatory state.

### 4.1. Chondrocytes, the Chondron and Matrix Turnover

The chondrocyte is the sole resident cell of articular cartilage and is responsible for synthesising, organising and maintaining the extracellular matrix on which the tissue’s mechanical function depends [1]. Chondrocytes occupy only a small fraction of tissue volume but sustain two dominant matrix systems. Type II collagen fibrils provide tensile and shear stiffness. Aggrecan’s sulphated glycosaminoglycan chains carry the fixed negative charge that generates osmotic swelling and contributes to compressive stiffness [1].

Cells and collagen are organised by depth: flattened cells and tangential fibrils at the surface, rounded cells and oblique fibrils in the middle zone, and cell columns with radial collagen in the deep zone. This architecture produces depth-dependent mechanical properties [1]. Each chondrocyte is immediately surrounded by a narrow pericellular matrix, rich in type VI collagen and the proteoglycan perlecan, which together with the enclosed cell constitutes the chondron, the basic structural and functional unit through which mechanical signals are transmitted to the cell [5,183,184].

The depth-dependent organisation of the tissue is functionally significant rather than merely descriptive. In the superficial zone, flattened chondrocytes, tangential collagen and lubricin support shear resistance and low-friction articulation. High fluid pressure and relatively low solid stress under load are consistent with a mechanically protective surface layer [1]. In the middle and deep zones, the increasingly perpendicular collagen anchored into the calcified cartilage resists compression and ties the tissue to the underlying bone, and the chondrocytes adopt the columnar arrangement and more oxidative, hypertrophy-prone phenotype that becomes important in disease [1].

The pericellular matrix is the immediate mechanical filter between zonally organised tissue and the cell. Its composition and intermediate stiffness regulate strain and fluid-flow transfer, while its receptors couple matrix to cell. The chondron is therefore an elementary mechanotransductive unit whose disruption in OA changes the signal received by the chondrocyte [5,183,184].

Matrix homeostasis reflects a continuous balance between synthesis and degradation. The principal catabolic enzymes are matrix metalloproteinases and ADAMTS aggrecanases. MMP-13 cleaves type II collagen; ADAMTS-5 is prominent in mouse tissue, whereas ADAMTS-4 and ADAMTS-5 contribute in human tissue. Tissue inhibitors of metalloproteinases constrain their activity [186,187,188]. Genetic deletion studies established the causal importance of these enzymes: mice lacking ADAMTS-5 activity are protected against aggrecan loss and cartilage degradation in models of osteoarthritis, and mice lacking MMP-13 are resistant to the collagen erosion that characterises the disease [186,187,188].

In healthy cartilage this proteolytic machinery serves normal turnover; in osteoarthritis its activity outstrips synthesis and inhibition, and the matrix is progressively destroyed. The composition and architecture of the cartilage matrix, the biology of cartilage homeostasis and its disturbance in disease, and the proteinases and their regulation have been reviewed comprehensively [178,179,180,181,182,185,189,190,193].

Causal evidence for ADAMTS-5 and MMP-13 is strongest in genetically modified mice, whereas the relative roles of ADAMTS-4 and ADAMTS-5 differ between mouse and human tissue [186,187,188]. This distinction matters when translating enzyme inhibition: protection in a surgical mouse model identifies mechanism within that model, not clinical efficacy in heterogeneous human OA.

### 4.2. Metabolic Stress, Impact Injury and Cell Survival

Cartilage is avascular, and the chondrocytes that inhabit it live in one of the most hypoxic environments in the body, with oxygen tensions falling to a few per cent and lower in the deep zone. The cells are correspondingly adapted to a largely glycolytic metabolism and depend on the hypoxia-inducible transcription factors to survive and function [194,195]. HIF-1α is essential for chondrocyte survival and matrix synthesis in the hypoxic interior, supporting glycolytic energy production and the autophagic housekeeping that maintains the cells [197].

Its relative HIF-2α, by contrast, drives a catabolic and hypertrophic programme, promoting the expression of matrix-degrading enzymes and the markers of terminal differentiation, and has been identified as a catabolic regulator of cartilage destruction in osteoarthritis [198,199]. The metabolic state of the chondrocyte its mitochondrial function, its handling of reactive oxygen species, and the balance between its anabolic and catabolic programmes is thus central to whether cartilage is maintained or destroyed, and metabolic dysregulation is increasingly recognised as a feature of the disease [194,195].

Two further cell-physiological processes shape the fate of cartilage with age and injury. The first is cellular senescence. With advancing age and after mechanical or oxidative injury, chondrocytes increasingly enter stable cell-cycle arrest. Their senescence-associated secretory phenotype comprises pro-inflammatory cytokines, chemokines and proteases that propagate dysfunction and degrade matrix [201,204]. The selective clearance of senescent cells has been shown to attenuate the development of post-traumatic osteoarthritis and to foster a more regenerative joint environment, identifying senescence as a causal contributor and a therapeutic target [202].

The second is autophagy, the regulated turnover of intracellular constituents, which is protective in healthy cartilage and declines with age; the loss of autophagy is associated with the cell death and matrix loss of ageing and osteoarthritic cartilage, and mitochondrial dysfunction features prominently in this decline [191,192,205]. The control of chondrocyte metabolism by oxygen tension and the hypoxia-inducible factors, the accumulation of senescent cells and oxidative damage with age, and the mitochondrial and autophagic mechanisms that maintain or fail the cell have been examined in depth [196,200,203,206,207,208,209].

Acute impact adds a distinct injury phase. Supraphysiological compression can rupture matrix, distort the plasma membrane and cause immediate or delayed chondrocyte death. Calcium overload, mitochondrial membrane dysfunction, reactive oxygen species (ROS), DNA damage and inflammatory signalling then propagate injury beyond the original contact site [191,192,193,194,195,196,197,198,199,200,201,202,203,204,205,206,207,208,209,226,237,238]. Evidence for these steps is mechanistically strong in cartilage explants and animal models; the timing and magnitude of the same pathways in human post-traumatic OA remain less directly resolved.

Autophagy, mitophagy and antioxidant capacity can buffer this response, but they decline with age and OA. Senescent cells and their secretory phenotype provide another amplification mechanism [201,202,203,204,205,206,207,208]. These processes prevent a purely mechanical interpretation of injury: an identical later gait pattern may have different consequences in tissue already primed by mitochondrial stress, senescence or inflammation.

### 4.3. Bone Cells and Osteochondral Crosstalk

Beneath the cartilage, the subchondral bone is governed by its own cellular triad and communicates with the cartilage across the osteochondral junction. The osteocyte, the most abundant bone cell, resides within the mineralised matrix in a lacuno-canalicular network ideally configured to sense the interstitial fluid flow produced by mechanical loading, and orchestrates the activity of the bone-forming osteoblasts and bone-resorbing osteoclasts [2]. Osteoclast differentiation and activity are controlled by receptor activator of nuclear factor kappa-B ligand (RANKL) and its decoy receptor osteoprotegerin. Their balance regulates resorption. The RANKL–OPG axis is altered in osteoarthritic subchondral bone and contributes to abnormal remodelling [211,212,214].

The osteocyte-derived Wnt antagonist sclerostin, the product of the SOST gene, modulates bone formation and is also expressed by articular chondrocytes, where increased expression appears to protect against cartilage degradation even as it influences subchondral sclerosis [144].

Cartilage and bone form a mechanically and biologically coupled osteochondral unit rather than two independent tissues [138]. Signals pass in both directions across the junction: transforming growth factor beta released during accelerated subchondral remodelling can recruit mesenchymal cells and disturb cartilage homeostasis, while altered cartilage modifies the mechanical environment of bone [220].

Therapeutic claims require anatomical and evidential qualification. A placebo-controlled phase 2a trial of denosumab reduced radiographic progression in erosive hand OA, not knee OA [218]. A 2025 knee study combined human tissue analyses, murine and canine models and a single-arm patient cohort; it reported RANK/TRAF6/FSTL1-related synovial effects and symptomatic improvement, but it was not a randomised knee-structure-modification trial [222]. These observations justify further testing; they do not establish structural disease modification in knee OA.

Sclerostin is similarly context-dependent: increased chondrocyte expression has been associated with protection, while genetic loss or pharmacological inhibition did not alter cartilage remodelling in some ageing and injury models [144,217]. Neither the RANKL nor sclerostin axis is therefore a simple, validated knee OA drug switch. The broader osteocyte, RANKL, Wnt–sclerostin and osteochondral-crosstalk literature supports biological plausibility while underscoring tissue- and disease-stage dependence [210,213,215,216,217,219,221,223].

### 4.4. Synovium, Innate Immunity and Systemic Modifiers

The synovium that lines the joint cavity is populated by two principal cell types: fibroblast-like synoviocytes, which produce the hyaluronic acid and lubricin of the synovial fluid and maintain the joint lining, and macrophage-like synoviocytes, which provide innate-immune surveillance [3]. Although osteoarthritis was long regarded as non-inflammatory, it is now clear that synovitis inflammation of the synovium is common, contributes to symptoms and structural progression, and is driven in large part by the innate immune response to damage-associated molecular patterns released from the breaking-down cartilage and bone [3,224].

Activated synovial cells secrete the pro-inflammatory cytokines interleukin-1 beta, tumour necrosis factor alpha and interleukin-6, which in turn drive chondrocytes toward catabolism by inducing matrix-degrading enzymes and suppressing matrix synthesis, establishing a self-reinforcing inflammatory circuit between synovium and cartilage [226,227]. This low-grade, innate-immune-driven inflammation is increasingly seen as a key mediator of the disease and a target for therapy, distinct from the adaptive autoimmune inflammation of rheumatoid arthritis [226].

The role of synovitis in the pathophysiology and symptoms of osteoarthritis, the contributions of fibroblast-like and macrophage-like synoviocytes and of innate immunity and the inflammasome, and the cytokine networks involved have been characterised across many studies [225,228,229,230,231,232,233,234,235,236].

Systemic metabolism can modify this local circuit. Adipose-derived mediators, insulin resistance and low-grade inflammation help explain why obesity is not only an increase in joint load [8,9,239,240]. The gut microbiome is an emerging modifier: germ-free mice developed less severe OA after destabilisation of the medial meniscus, which supports causality within that animal model [241]. Human knee studies report microbial or lipopolysaccharide-related differences and associations with symptoms or imaging, but heterogeneous methods, contamination risk and confounding preclude a claim that dysbiosis causes human OA [242].

### 4.5. Fibroblasts, Progenitors and Homeostatic Signalling

The ligaments and tendons of the knee are maintained by fibroblasts aligned along the collagen fibres, which sense tensile load and remodel the matrix accordingly, adjusting collagen synthesis and crosslinking to the prevailing mechanical demand. Where these tissues insert into bone, the enthesis forms a graded transition through fibrocartilage that smooths the transfer of stress between compliant tendon or ligament and stiff bone; this gradient is mechanically optimised but is also a site of stress concentration and a common locus of injury and degeneration. Mesenchymal stromal and progenitor populations occur in synovium, subchondral marrow and the superficial cartilage zone.

They participate in repair and motivate cell-based regenerative strategies, although adult cartilage has limited intrinsic healing capacity [1].

The behaviour of all these cells is coordinated by an interacting network of signalling pathways whose balance determines whether tissue is maintained or destroyed. Transforming growth factor beta and the bone morphogenetic proteins, signalling through SMAD transcription factors, generally promote matrix synthesis and the maintenance of the chondrocyte phenotype, although their effects are strongly context-dependent and can become pathological when chronically dysregulated [243]. Wnt/beta-catenin signalling promotes chondrocyte hypertrophy, catabolism and the differentiation programme that, when inappropriately reactivated in articular cartilage, contributes to degeneration; its careful regulation is essential, as both excessive and deficient Wnt activity are deleterious to the joint [243].

The Indian hedgehog and parathyroid-hormone-related-protein feedback loop that controls the orderly progression of chondrocyte hypertrophy in the growth plate is partially recapitulated in osteoarthritic cartilage, where chondrocytes lose their stable articular phenotype and advance toward hypertrophy and calcification [243]. Fibroblast growth factor signalling exerts dual effects, with FGF-2 acting largely catabolically and tethered within the pericellular matrix where it serves as a mechanotransducer, and insulin-like growth factor 1 acting anabolically [244,245]. Nuclear factor kappa-B integrates inflammatory, mechanical and oxidative signals and drives expression of matrix-degrading enzymes [244]. Its context-dependent interaction with YAP/TAZ links inflammatory and mechanical regulation of cartilage fate.

With age and disease these context-dependent modules shift collectively toward catabolism, hypertrophy and inflammation, tilting the homeostatic balance toward matrix destruction. The transforming-growth-factor-beta, bone-morphogenetic-protein, Wnt, hedgehog, fibroblast-growth-factor and integrin signalling pathways and their roles in cartilage and subchondral bone in health and osteoarthritis have been reviewed in detail [246,247,248,249,250,251,252,253,254,255].

Transforming growth factor beta, Wnt, fibroblast growth factor, nuclear factor kappa-B (NF-κB) and related networks are dose-, time-, tissue- and disease-stage dependent [243,244,245,246,247,248,249,250,251,252,253,254,255]. Describing one pathway as uniformly anabolic or catabolic obscures this dependence. The useful mechanistic question is which cell population, mechanical regimen and inflammatory background produce the reported response.

## 5. Mechanobiology and Mechanotransduction

Mechanotransduction connects tissue deformation to biochemical signalling and gene regulation. Relevant sensors include ion channels, primary cilia, integrins, focal adhesions, the actomyosin cytoskeleton and nuclear mechanosensitive relays. These systems overlap and compensate; their output depends on stimulus magnitude, rate, duration, loading history, osmolarity, matrix stiffness, cell state and inflammation [7,237,244,245,256,257,258,259,260,261,262,263,264,265,266,267,268,269,270,271,272,273,274,275,276,277,278].

### 5.1. TRPV4 and PIEZO1/2: Overlapping, Context-Dependent Pathways

TRPV4 is a polymodal, calcium-permeable channel that responds to osmotic, thermal and mechanically coupled signals in chondrocytes [261,262,263,264,265,266]. Dynamic compression near 10% strain in a canonical explant study engaged TRPV4-dependent calcium signalling and matrix synthesis [261], but this experimental regimen is not a universal physiological threshold. TRPV4 may be activated indirectly through osmotic or signalling intermediates rather than by membrane stretch alone [265]. Its effect also differs by cell type and OA context: global TRPV4 deficiency accelerated age-related pathology, whereas inducible cartilage-specific loss reduced age-associated OA and did not protect against surgical post-traumatic OA [264,279]. TRPV4 therefore cannot be described as uniformly protective.

PIEZO1 and PIEZO2 are directly mechanically activated cation channels with a membrane-curving trimeric architecture [256,257,258,259]. In vitro high-strain compression of chondrocytes engaged PIEZO1/2-dependent calcium entry and cell injury [260,265]. Chondrocyte-specific deletion of both channels reduced structural damage and pain after joint injury in mice, providing causal evidence in that model [237]. Human OA cartilage shows altered PIEZO1 expression, but this remains associative [267,268,280]. Inflammatory interleukin-1 signalling can increase PIEZO1 expression and mechanically sensitise chondrocytes, creating a feed-forward interaction between inflammation and mechanics [280].

A phenomenological two-state relation can summarise channel recruitment as a function of membrane tension γ,

(3) *Popen* = 1/{1 + *exp*[−(*γ* − *γ*50)/*sγ*]}

where Popen is open probability, γ50 is the half-activation tension and sγ controls slope [281]. Equation (3) is a compact local approximation, not a conserved chondrocyte constant: apparent sensitivity changes with membrane composition, cytoskeletal coupling, channel density, subcellular localisation and the loading method. It should not be converted directly into a tissue-strain threshold without a validated membrane-tension model.

### 5.2. Primary Cilia as Mechano-Chemical Signalling Hubs

Most chondrocytes possess one primary cilium. Beyond serving as a site for mechanically evoked calcium signalling, the cilium organises polycystin/TRP-family channels, integrin-associated receptors and hedgehog signalling and can influence cyclic-AMP and extracellular ATP pathways [271,272,273]. Ciliary length and orientation change with tissue zone, loading and disease, potentially altering which signal reaches the cell body.

Evidence that ciliary disruption changes chondrocyte mechanosensitivity is causal in cell and animal models, but individual ciliary components have pleiotropic developmental roles. A phenotype after cilium ablation cannot automatically be assigned to mechanical calcium entry. Distinguishing acute sensory function from developmental, trafficking and transcriptional effects remains a key experimental need.

### 5.3. Integrin–Cytoskeleton–Nucleus Coupling and YAP/TAZ

Integrins bind matrix ligands and assemble focal adhesions containing talin, vinculin, focal-adhesion kinase and associated signalling proteins. Force-dependent adhesion maturation activates Rho-family GTPases; RhoA–ROCK increases myosin light-chain phosphorylation, actomyosin contractility and stress-fibre assembly. Cytoskeletal tension is transmitted to the nucleus through the linker of nucleoskeleton and cytoskeleton (LINC) complex and changes nuclear shape, chromatin accessibility and transcriptional cofactor localisation [7,274,275,276,277,278].

The LINC complex is what makes this final step mechanical rather than purely chemical. SUN-domain proteins in the inner nuclear membrane bind the nuclear lamina, while outer-membrane nesprins bind actin, microtubule motors and intermediate filaments, so that cytoskeletal force is carried as a physical continuum to the lamina and chromatin rather than through diffusible messengers alone. Disrupting this bridge alters nuclear stiffness, lamin tension and the nuclear access of mechanosensitive regulators, giving mechanical load a direct structural route to gene expression in chondrocytes [7,274,275,276,277,278].

YAP and TAZ are prominent outputs of this chain. Matrix stiffness, cell spreading and actomyosin tension can promote their nuclear localisation, whereas Hippo-pathway phosphorylation favours cytoplasmic retention [274,275]. In cartilage, however, YAP/TAZ effects depend on developmental stage, zone and inflammatory context, and crosstalk with NF-κB is not a simple reciprocal switch [244,277]. Much of the causal Rho/actin-to-YAP/TAZ sequence was established in reductionist cell systems; direct quantitative mapping in loaded human cartilage remains incomplete.

### 5.4. Calcium Dynamics and the Load-Response Continuum

Mechanical stimulation produces calcium transients whose amplitude, frequency, duration and subcellular location encode more information than peak concentration alone [261,265,280]. Influx through TRPV4 or PIEZO channels interacts with release and reuptake by intracellular stores. Candidate decoding modules include calmodulin/CaMK, calcineurin–NFAT, MAPK/ERK and calcium-sensitive transcriptional regulators; inflammation adds NF-κB and p38-dependent reprogramming. The coupling from a measured calcium waveform to a specific matrix-gene response has nevertheless rarely been calibrated in situ.

Moderate dynamic loading often supports matrix synthesis, whereas prolonged static loading or acute high-magnitude impact can favour catabolism, ROS generation, mitochondrial dysfunction and apoptosis [205,237,261,282,283]. These are tendencies across experimental regimes, not a binary threshold shared by every joint.

### 5.5. Single-Cell Mechanics and Strain Transfer

Micropipette aspiration and atomic force microscopy (AFM) estimate chondrocyte and pericellular matrix properties from pressure–deformation or force–indentation data [165,166,167,168,169,170,171,172,173,174,175,176,177,284,285]. The corrected elastic aspiration relation, standard-linear-solid relaxation law and Hertz/Sneddon indentation equations are provided in Appendix A. Each inversion assumes a geometry, boundary condition and constitutive behaviour; reported moduli therefore depend on probe scale, indentation depth, adhesion, osmolality and fit window.

These measurements established the mechanical hierarchy of the chondron that governs how tissue strain reaches the cell. The chondrocyte itself is very soft, with a Young’s modulus of roughly half a kilopascal, while the pericellular matrix is far stiffer and the extracellular matrix stiffer still. Micropipette aspiration of isolated chondrons gave pericellular-matrix moduli of approximately 23–24 kPa in canine cartilage and 62–69 kPa in human cartilage [169,170]. Values varied by species and method, and the modulus decreased as the pericellular matrix degraded in OA.

Because the soft cell is embedded in a stiffer pericellular matrix within a much stiffer extracellular matrix, the strain transmitted to the cell depends sharply on the ratio of these moduli, a relationship made quantitative by biphasic and multiscale finite element models of the chondron [6,175]. Guilak and Mow’s biphasic finite element model showed how property mismatches among cell, pericellular matrix and extracellular matrix concentrate or attenuate strain, fluid pressure and flow at the cell. Confocal measurements of chondrocyte deformation in loaded cartilage confirmed the predicted strain transfer [6,177].

The mechanobiological implication is direct: the degradation of the pericellular matrix in early osteoarthritis alters the mechanical signal that the chondrocyte receives, so that the same tissue-scale load is transduced into an abnormal cellular stimulus, coupling matrix damage to disordered mechanotransduction in a feed-forward loop [5]. The micropipette-aspiration and atomic-force-microscopy techniques, the viscoelastic and osmotic properties of the chondrocyte and its nucleus, and the modelling of the pericellular microenvironment and of strain transfer to the cell have been developed in a substantial methodological literature [166,168,171,172,173,174,176,284,286].

### 5.6. Exercise, Compensation and Mechanosensor Comparison

The principle that moderate loading maintains tissue while disuse and overload degrade it extends across the tissues of the joint and connects the cellular mechanobiology to whole-organism physiology. In cartilage, moderate physical activity supports matrix turnover and is broadly chondroprotective, an effect attributable in part to the augmentation of mechanosensitive-channel-mediated anabolic signalling under physiological loading, whereas immobilisation leads to cartilage thinning and proteoglycan loss [261,283]. In bone, the same logic is formalised by the mechanostat of Section 3, in which the osteocyte network senses load-induced fluid flow and adjusts bone mass to keep strain within a physiological window [2].

The therapeutic corollary is that controlled mechanical loading through exercise, rehabilitation, and the mechanical conditioning of engineered tissues is itself a biological intervention, capable of steering cells toward matrix synthesis, a theme taken up in the discussion of tissue engineering in the next section.

Adaptation also implies compensation. Blocking one mechanosensor can redistribute signalling through other channels, integrins, cilia, purinergic receptors or cytoskeletal pathways. Chronic genetic deletion may therefore produce a different phenotype from acute pharmacological inhibition. Table 4 compares the major sensors using evidence source and interpretive limitation rather than assigning a single universal function.

## 6. Osteoarthritis as a Whole-Joint Mechanobiological Disease

### 6.1. Burden, Phenotypes, Sex and Grading

OA integrates cartilage degradation, meniscal and ligament pathology, synovitis, osteophyte formation and subchondral remodelling [4,8,9,10,11]. The Global Burden of Disease analysis estimated approximately 595 million people living with OA in 2020, with knee OA the dominant site and substantial further growth projected [8,9]. This epidemiological scale makes mechanobiological precision clinically relevant, but population burden does not imply one molecular mechanism; OA includes post-traumatic, metabolic, ageing-associated and mechanically malaligned phenotypes [11,287].

Women experience a higher burden of knee OA, particularly later in life, but sex and gender are often conflated. Sex-related anatomy, hormones, metabolism, immune biology and injury patterns may interact with gendered differences in exposure, occupation, sport, care and social determinants [8,288]. Preclinical experiments should report and power for sex, and human models should avoid treating sex as a nuisance covariate when it may alter geometry, material properties or inflammatory response.

The Kellgren–Lawrence (KL) grade remains useful for radiographic cohort stratification [289], but it is an ordinal late-structure measure. Joint-space width is an indirect surrogate influenced by cartilage and meniscus, early molecular and compositional changes are poorly captured, and reader variability can be material. A computational model that separates KL groups has therefore not necessarily predicted early biological progression or patient-important outcomes.

### 6.2. Mechanical Risk, Post-Traumatic OA and Feedback

The mechanical aetiology of knee osteoarthritis is evident in its risk factors. Obesity is among the strongest, carrying a pooled odds ratio for knee osteoarthritis of roughly two and a half to three and rising steeply at the highest body weights, through a combination of increased joint loading and metabolic and inflammatory effects [239,240]. Malalignment of the limb, which concentrates load on one compartment, is a powerful driver of structural progression once disease is established, and the loss of meniscal function through tearing or resection sharply elevates cartilage stress and risk [290].

The clearest demonstration that mechanics can initiate the disease is post-traumatic osteoarthritis, in which a defined mechanical injury most commonly rupture of the anterior cruciate ligament, often with associated meniscal damage sets in train a process that culminates in osteoarthritis years later [291]. Risk after anterior cruciate ligament injury increases with time. Syntheses report post-traumatic OA in approximately 38–52% of injured knees over one to two decades, with time-stratified estimates rising from about 11% at five years to more than 50% at twenty years [292,293].

Post-traumatic osteoarthritis is of particular value to the mechanobiologist because its initiating event is known and datable, making it the natural setting in which to study, and to model, the progression from mechanical insult to established disease. Large cohort syntheses cover knee OA epidemiology, risk factors, meniscal and ligament injury, synovitis and clinical phenotypes [287,289,294,295,296,297,298]. Structural severity is conventionally graded with the Kellgren–Lawrence system.

The cascade that links the initiating injury to eventual osteoarthritis is itself mechanobiological and unfolds over distinct phases. The acute injury delivers a supraphysiological impact to the cartilage that kills chondrocytes and damages the collagen network directly, and it triggers an immediate burst of inflammatory cytokines and matrix-degrading enzymes within the joint, an early biological insult superimposed on the mechanical one [226,238].

The rupture of the anterior cruciate ligament then alters the kinematics and load distribution of the joint chronically, shifting the location of contact and the pattern of cartilage loading even after surgical reconstruction, so that regions of cartilage unaccustomed to high load now bear it [291]. The combination of an initial biological injury, a persistent inflammatory environment, and an altered mechanical environment establishes the conditions for the slow degeneration that follows, and it is the reason that reconstruction of the ligament, while it restores gross stability, does not reliably prevent osteoarthritis [292,293].

For the modeller this phased cascade is an opportunity: because the timing of the initiating event is known, the subsequent change in joint mechanics can be simulated and coupled to a degeneration model, making post-traumatic osteoarthritis the principal proving ground for predictive, subject-specific models of disease progression [80].

**Figure 3:**
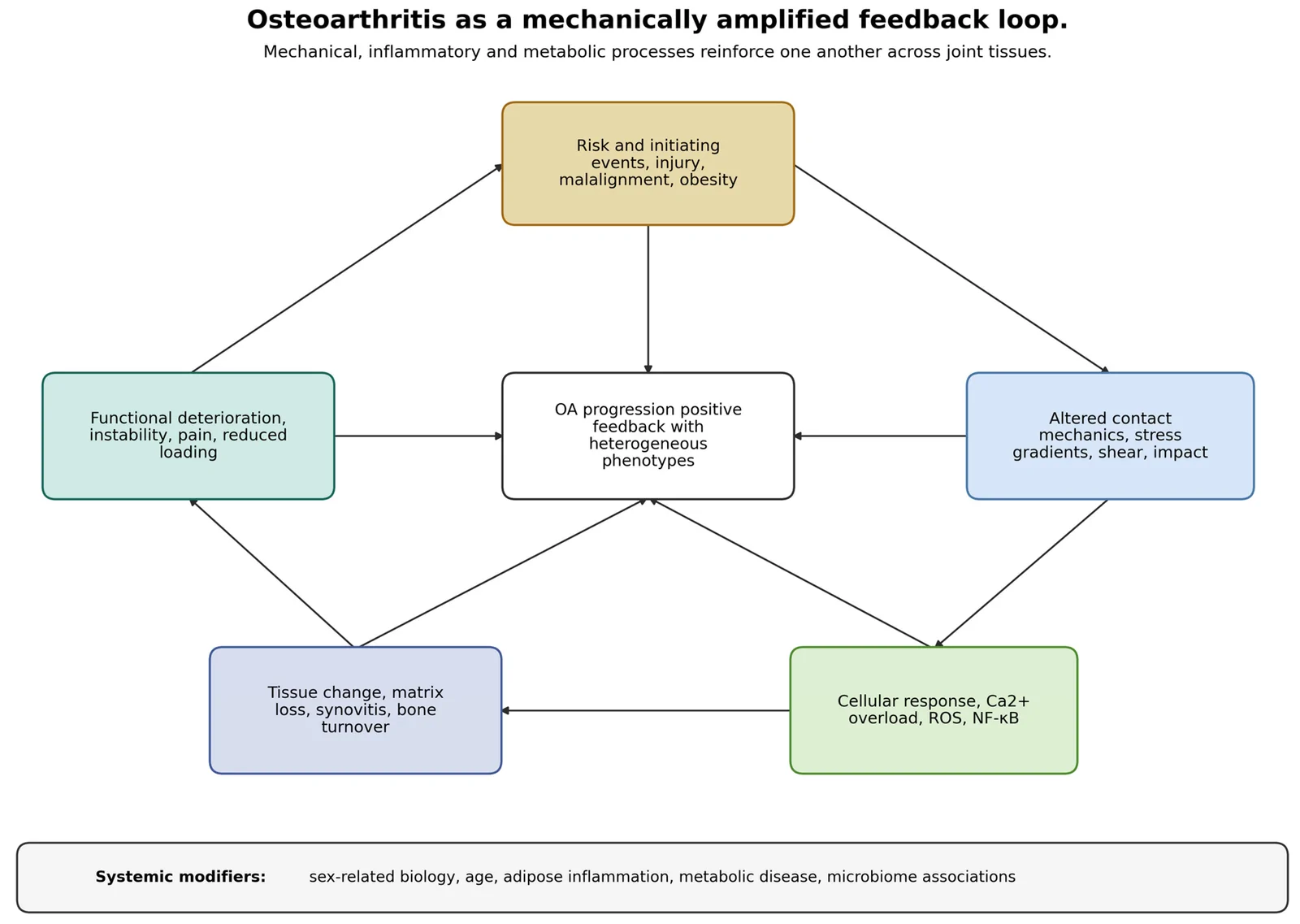
Osteoarthritis as a mechanically amplified feedback loop. Initiating risks alter contact mechanics; cellular stress and inflammatory responses drive tissue change; and the resulting instability, pain and activity adaptation feed back to joint loading. Systemic factors modify several links. The diagram does not imply that every patient follows the same sequence.

Obesity adds both load and systemic inflammatory or metabolic exposure; malalignment redistributes compartmental load; and meniscal or ligament injury changes contact location and stability [239,240,290,291,292,293,294,295,296]. After trauma, immediate matrix rupture and chondrocyte death coexist with sustained kinematic change and synovial inflammation. Figure 3 depicts OA progression as positive feedback rather than a linear cascade and includes sex-related biology, age, metabolism and microbiome associations as modifiers rather than proven single causes.

### 6.3. Mechanistic Models of OA Progression

Progression models couple FE-derived mechanical fields to rules for collagen damage, proteoglycan loss, cytokine transport or tissue adaptation [80,238,282,283,298,299,300,301,302,303,304,305,306,307,308,309,310,311,312]. Wilson et al. examined candidate causes of mechanically induced collagen damage [300], and Hosseini et al. simulated initiation and progression using mechanical failure criteria [299]. These studies support mechanistic plausibility and sensitivity analysis, but neither constitutes longitudinal patient-level validation.

Mononen and colleagues used Osteoarthritis Initiative data to relate subject-specific loading and FE outputs to spatial cartilage degeneration [80,298]. This was an important bridge to human cohorts, yet the outcomes remained sensitive to estimated loads, template geometry, material assumptions and radiographic grading. Orozco et al. predicted proteoglycan loss after mechanical injury and dynamic loading in cartilage explants [303], while Eskelinen et al. added pro-inflammatory cytokine transport and signalling [238]. Their ex vivo validation is stronger for local biology but does not establish whole-knee clinical prognosis.

Table 5 distinguishes mechanistic validation, surrogate fidelity and clinical utility. Growth and constrained-mixture theories provide rigorous language for matrix production, removal and residual stress [304,305,306,307], while mechanically conditioned tissue engineering demonstrates that the same pathways can be used for repair [283,311]. The central unresolved issue is parameter identification across time: several different damage rules can reproduce the same structural endpoint.

### 6.4. Machine Learning, Surrogates and Digital-Twin Readiness

Machine learning can accelerate segmentation, joint-load estimation and FE emulation [313,314,317,318,319,320,321,322,323,324,325,326,327,328]. Neural and graph-network surrogates can reproduce a parent simulation quickly, including altered conditions such as meniscal extrusion [313,314,323]. Their accuracy is bounded by the training distribution and by the parent model: a surrogate can be numerically faithful while inheriting incorrect constitutive assumptions or unvalidated boundary conditions. External testing, calibration and uncertainty reporting are therefore more important than speed alone.

The term digital twin should be used with maturity labels. A static patient-specific model is a digital model; one-way updating from patient data is often called a digital shadow; a digital twin should support repeated state updating and clinically relevant counterfactual prediction [315,318,329]. Hoyer et al. described foundations based on cross-sectional qMRI biomarkers [315]. Jayakumar et al. evaluated an AI decision aid that used PROMs and clinical data to personalise arthroplasty benefit–risk estimates [316]. These are important but distinct achievements; neither is a prospectively validated, continuously updated multiscale knee twin.

**Figure 4:**
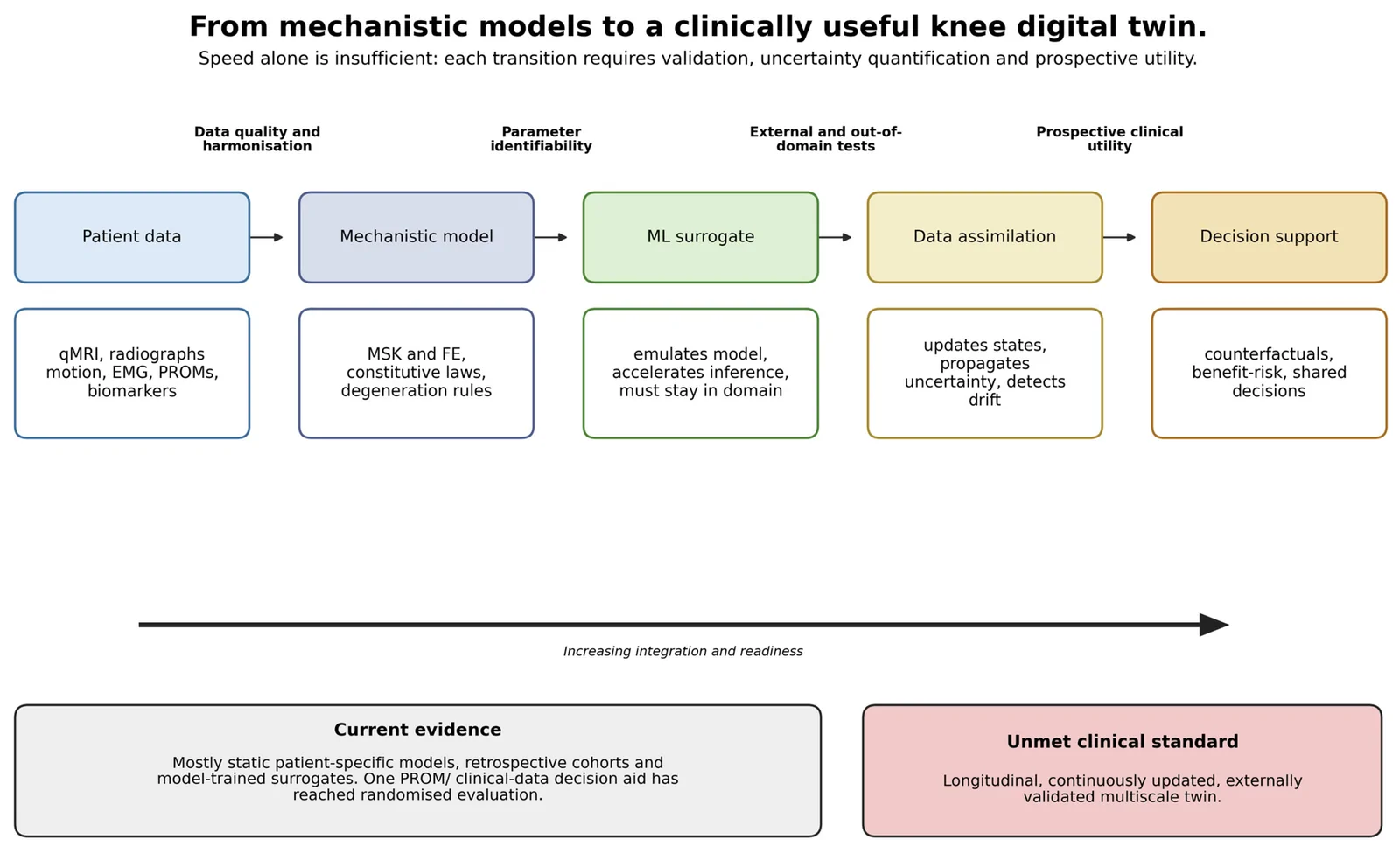
Integration of mechanistic models, machine-learning surrogates and data assimilation toward clinical decision support. Vertical bars mark validation gates. Current knee applications are predominantly static models, retrospective analyses or model-trained surrogates; a continuously updated, externally validated multiscale twin remains an unmet standard.

Major barriers include harmonised longitudinal imaging and load data, parameter identifiability, propagation of uncertainty across scales, model drift, external validation, interoperability, interpretability, privacy, bias, regulatory classification and integration into clinical workflow [315,316,318,326,327,328,329,330]. Figure 4 shows the required validation gates and distinguishes current evidence from the unmet clinical standard.

Regulatory deployment raises challenges beyond technical validation. A predictive knee twin used to guide care would, in most jurisdictions, be regulated as software as a medical device, and an adaptive or continuously updated model fits poorly with approval frameworks designed for fixed algorithms. Regulators have begun to address this through mechanisms such as predetermined change-control plans and good machine-learning practice, and through medical-device and artificial-intelligence rules that place high-risk clinical AI under added obligations for risk management, transparency and human oversight. Practical requirements include prospective rather than retrospective validation, version control of both model and training data, auditable and interpretable outputs, protection of longitudinal patient data, post-deployment monitoring for performance drift, and evidence of clinical utility and equitable performance across sites and populations. These demands, as much as any modelling limitation, currently separate credible research prototypes from tools ready for routine clinical use [315,316,318,326,327,328,329,330].

### 6.5. Therapeutic Implications: Evidence and Qualification

Mechanobiological pathways are attractive because they connect an exposure to a modifiable response, but the therapeutic evidence is uneven. Table 6 separates target rationale from the strongest evidence and clinical readiness. In particular, mouse protection after channel deletion, a human-tissue association and a trial in hand OA are not interchangeable forms of support for knee OA treatment.

## 7. Synthesis, Knowledge Gaps and Research Priorities

### 7.1. What the Cross-Scale Framework Changes

The central synthesis is not that every scale can already be predicted. It is that each transfer can be specified and tested. Musculoskeletal models estimate force; tissue models transform force into local fields; chondron models estimate membrane, osmotic and cytoskeletal stimuli; molecular experiments identify response pathways; and turnover models feed altered properties back into mechanics [5,6,32,74,84,175,256,304,305]. The conceptual advance is the accompanying validation ladder: telemetry cannot validate gene expression, and an accurate calcium trace cannot rescue an incorrect contact-pressure field.

This framework also clarifies evidence maturity. Whole-joint loads are directly measured in a small TKR population; material parameters vary by tissue and protocol; cellular mechanisms are frequently causal in mouse or in vitro systems; and longitudinal human coupling remains sparse. A credible multiscale prediction must propagate these uncertainties instead of presenting a single deterministic disease trajectory.

### 7.2. Current Knowledge Gaps and Priorities

1. Human cross-scale calibration. Acquire longitudinal combinations of motion, muscle activation, quantitative imaging, synovial or blood biomarkers and outcomes in the same individuals, including early and post-traumatic disease.
2. Context-resolved mechanotransduction. Map calcium waveform, channel localisation and downstream gene response across load amplitude, rate, duration, tissue zone, age, sex, inflammatory state and OA stage rather than relying on one nominal threshold.
3. Parameter identifiability and uncertainty. Use multi-modal experiments, Bayesian or ensemble calibration and sensitivity analysis to distinguish parameters that can be estimated from those that should remain distributions.
4. Validation beyond KL grade. Adopt compositional MRI, three-dimensional morphology, symptoms, function and molecular endpoints alongside radiographs, with independent and prospective cohorts.
5. Generalisability and inclusion. Report sex, age, ancestry, body composition, disease phenotype and loading exposure; test model drift and fairness across sites and acquisition systems.
6. Translational perturbation studies. Test TRPV4, PIEZO1/2, RANKL, sclerostin, NF-κB and YAP/TAZ interventions with tissue-specific delivery, dose timing, safety and human-relevant mechanical regimens before claiming disease-modifying potential.
7. Digital-twin standards. Define minimum requirements for repeated data assimilation, uncertainty, external validation, clinical utility, interoperability, privacy and regulatory monitoring; reserve the term digital twin for systems that meet the declared maturity level.

These priorities suggest a phased roadmap. In the near term (roughly 0–2 years), the field can standardise reporting and validation endpoints (priorities 4 and 5), adopt uncertainty-aware calibration in existing models (priority 3), and agree minimum maturity criteria for the term digital twin (priority 7). In the medium term (about 3–5 years), coordinated multi-site cohorts can begin the longitudinal, multi-modal data collection needed for genuine cross-scale calibration (priority 1) and context-resolved mechanotransduction mapping (priority 2), while tissue-specific perturbation studies test the leading molecular targets (priority 6). Over 5–10 years, these strands can converge on prospectively validated, uncertainty-aware multiscale models whose clinical utility is tested in diverse populations. This timeline is indicative rather than prescriptive; progress will depend on data-sharing infrastructure, funding and regulatory alignment as much as on modelling advances.

## 8. Conclusions

The knee is best understood as a coupled mechanobiological organ, but the value of a multiscale review lies in disciplined connection rather than breadth alone. This review’s specific contribution is a transmission-and-validation framework that follows force to tissue fields, cellular signals and matrix turnover while identifying assumptions, evidence source and uncertainty at each interface. The same framework explains OA as feedback among mechanics, cartilage, bone, synovium and systemic context rather than as a single linear pathway.

Several conclusions follow. TRPV4 and PIEZO1/2 have context-dependent and partly overlapping roles; RANKL, sclerostin, NF-κB and YAP/TAZ remain qualified therapeutic hypotheses rather than established knee OA targets; and current machine-learning or digital-twin studies do not yet provide a continuously updated, prospectively validated multiscale model for routine care. Progress now depends less on adding another mechanism than on measuring the links between scales, quantifying uncertainty and testing whether integrated models improve decisions in diverse patients.

Ultimately, the promise of knee mechanobiology lies in connection rather than accumulation. If the transfers between joint loading, tissue mechanics, cellular signalling and matrix turnover can be measured, calibrated and validated with honest uncertainty, integrated mechanobiological models could move osteoarthritis care from reactive, radiograph-based staging toward earlier, mechanistically grounded and individualised intervention. Realising that potential will require sustained collaboration across biomechanics, cell biology, computation and clinical medicine, but it offers a credible path to better outcomes for the millions of people affected by knee osteoarthritis.

## Figures and Tables

**Table 1:** Representative mean peak resultant tibiofemoral forces measured with instrumented knee replacements during activities of daily living [33]. Values are percentages of body weight and are descriptive of five TKR recipients, not universal thresholds for native knees.

Activity	Mean Peak Force (% BW)	Interpretive Limitation
Stair descent	346	High demand; cadence, handrail use and implant geometry affect the peak.
Stair ascent	316	Strongly influenced by quadriceps force and co-contraction.
Level walking	261	Two stance-phase peaks; speed and individual strategy vary.
One-leg stance	259	Balance strategy and frontal-plane moment influence load sharing.
Deep knee bend	253	Flexion angle and support strategy influence contact location.
Sit-to-stand	246	Seat height and forward trunk inclination change the demand.
Stand-to-sit	225	Eccentric control and movement speed are relevant.
Two-leg stance	107	Static value; medial–lateral distribution remains subject specific.

**Table 2:** Constitutive models used for knee tissues and interfaces. Model choice should follow the biological quantity of interest and the available validation data rather than computational convenience alone.

Tissue/Interface	Representative Model	Representation and Output	Strength	Limitation/Validation Need
Articular cartilage	Biphasic/poroelastic	Solid matrix + interstitial fluid; stress relaxation, creep and fluid pressure.	Mechanistically captures flow-dependent behaviour and load support.	Linear forms omit fibril anisotropy, fixed charge and large strain; test in compression/indentation.
Articular cartilage	Triphasic/multiphasic	Solid, fluid and mobile ions; swelling, osmotic pressure and electrokinetics.	Links proteoglycan fixed charge to mechanics and transport.	Many coupled parameters; bathing conditions and charge density must be measured.
Cartilage/meniscus	Fibril-reinforced poroviscoelastic	Non-fibrillar matrix, tensile collagen network, fluid flow and optional intrinsic fibril viscosity.	Separates proteoglycan and collagen contributions; supports depth dependence.	Parameter identifiability and fibre architecture are limiting; validate against several test modes.
Meniscus	Transversely isotropic or fibre-reinforced poroelastic	Circumferential fibres, radial ties and fluid phase; extrusion and hoop stress.	Represents architecture-driven load distribution.	Root constraints, degeneration and regional properties strongly affect predictions.
Ligament/tendon	QLV or anisotropic hyperelastic/HGO-type	Toe region, fibre recruitment, relaxation and directional stiffness.	Suitable for large tensile deformation and fibre dispersion.	Parameters depend on preconditioning, strain rate and reference configuration.
Subchondral bone	Elastic/poroelastic + adaptation law	Apparent stiffness, fluid flow and density or damage evolution.	Couples support mechanics to remodelling.	Mechanostat thresholds are phenomenological and not universal.
Synovial interface	Non-Newtonian fluid + boundary/biphasic lubrication	Shear-thinning fluid film, lubricin/HA boundary layer and interstitial-fluid support.	Explains very low friction across regimes.	Surface chemistry, roughness and time-dependent pressurisation require separate measurements.

**Table 3:** Representative mechanical parameters and reported variability. Ranges are deliberately broad and should be replaced by specimen-, region- and protocol-specific measurements whenever the output is sensitive to the parameter [84,85,87,88,89,90,91,92,93,94,95,96,97,98,99,100,101,102,105,106,107,108,111,125,126,127,128,129,130,131,132,133,134,135,136,137,139,158,165,166,167,168,169,170,171,172,173,174,175,176,177].

Material	Parameter	Indicative Range	Major Sources of Variability	Typical Use
Cartilage	Aggregate/equilibrium modulus	≈0.1–2 MPa	Depth, site, age, OA grade, confinement and fit model.	Consolidation and equilibrium compression.
Cartilage	Hydraulic permeability	≈0.1–10 × 10^−15^ m^4^/(N·s)	Strain, fixed charge, temperature, specimen thickness and inverse-fit method.	Fluid pressure, relaxation and transport.
Cartilage	Fluid volume fraction	≈65–85%	Depth, disease, hydration and measurement method.	Mixture mass balance and pressurisation.
Cartilage	Tensile modulus	≈5–25 MPa	Surface orientation, depth, strain rate and collagen integrity.	Fibril reinforcement and tensile damage.
Meniscus	Circumferential tensile modulus	≈100–300 MPa	Region, fibre direction, degeneration and testing rate.	Hoop-stress transmission and extrusion.
Ligament	Linear-region tangent modulus	≈100–300 MPa	Ligament, bundle, age, preconditioning, strain rate and reference length.	Restraint, laxity and fibre recruitment.
Trabecular bone	Apparent Young’s modulus	≈0.05–2 GPa	Density, anisotropy, site and imaging-to-property mapping.	Osteochondral support and remodelling.
Chondrocyte	Young’s modulus	≈0.3–1 kPa	Isolation, zone, osmolality, probe geometry and viscoelastic fit.	Cell deformation and membrane strain.
Pericellular matrix	Young’s modulus	≈20–70 kPa	Species, disease, zone and aspiration/indentation model.	Mechanical filtering within the chondron.
Synovial fluid	Apparent viscosity	≈0.01–1 Pa·s	Shear rate, HA molecular weight, inflammation and temperature.	Fluid-film and mixed lubrication.

**Table 4:** Major mechanosensors and downstream pathways in joint tissues. Experimental loading regimens are examples from specific studies, not universal physiological or injury thresholds.

Sensor/Structure	Stimulus or Representative Regimen	Principal Downstream Events	Strongest Evidence Represented	Critical Qualification
TRPV4	Osmotic change; dynamic compression (≈10% in a canonical explant protocol).	Ca^2+^ transients; context-dependent matrix synthesis and inflammatory modulation.	In vitro/ex vivo perturbation; global and cartilage-specific mouse genetics.	Polymodal and often indirectly mechanically coupled; age, tissue and OA-model effects conflict.
PIEZO1/2	Membrane tension; high-strain cell compression (≈50% in a canonical injury experiment).	Rapid Ca^2+^ entry; ROS, apoptosis and catabolic signalling under injurious conditions.	In vitro electrophysiology; chondrocyte-specific double-knockout mouse PTOA.	Not exclusively pathological; expression and sensitivity are increased by inflammatory state.
Primary cilium	Compression, fluid flow, osmotic change and matrix deformation.	Channel/receptor organisation, Ca^2+^/cAMP/ATP signals, hedgehog-related pathways.	Cell and animal perturbation.	Ablation also changes development, trafficking and cell phenotype.
Integrins/focal adhesions	Matrix strain, ligand binding, shear and substrate stiffness.	FAK/Src, RhoA–ROCK, actomyosin tension and MAPK signalling.	In vitro perturbation; tissue/explant studies.	Ligand, cell shape and matrix composition strongly condition the response.
Actin–LINC–nucleus	Cytoskeletal tension and nuclear deformation.	Nuclear mechanics, chromatin organisation and transcriptional access.	General mechanobiology; emerging cartilage-specific evidence.	Quantitative in situ transfer functions remain poorly identified.
YAP/TAZ	Matrix stiffness, spreading, Rho/actomyosin tension and Hippo signalling.	Nuclear co-activation of context-dependent transcriptional programmes.	Causal cell studies; animal and cartilage associations.	Effects vary by developmental stage, zone, duration and inflammation.
Osteocyte network	Load-induced canalicular fluid flow and matrix strain.	Ca^2+^/NO/PGE_2_; RANKL/OPG and sclerostin-linked remodelling.	Animal genetics and bone-loading experiments.	Mechanostat set-points are tissue- and history-dependent, not fixed constants.

**Table 5:** Representative computational approaches for knee OA and their validation status. FE, finite element; OAI, Osteoarthritis Initiative; qMRI, quantitative magnetic resonance imaging; PROM, patient-reported outcome measure.

Approach/Study	Mechanism or Output	Validation Represented	Strength	Limitation/Readiness
Wilson et al., 2006 [300]	Collagen damage from local mechanical stimuli.	Comparison with experimental cartilage behaviour.	Tests competing damage mechanisms.	Proof of principle; no longitudinal human prediction.
Hosseini et al., 2014 [299]	Numerical initiation and progression of cartilage damage.	Parameterised from the experimental literature.	Explicit iterative damage–mechanics coupling.	Threshold and parameter sensitivity; no clinical validation.
Mononen et al., 2016/2018 [80,298]	Subject-specific collagen degeneration and proteoglycan loss.	Retrospective OAI structural/radiographic data.	Links gait-scale loading to human spatial progression.	Estimated loads, simplified geometry and KL-type endpoints limit inference.
Orozco et al., 2018 [303]	Mechanical injury + dynamic loading → proteoglycan loss.	Bovine cartilage explants over time.	Direct local mechanobiological validation.	Ex vivo tissue and short horizon; not a whole-joint clinical model.
Eskelinen et al., 2020 [238]	Cytokine diffusion plus mechanical damage stimulus.	Injured cartilage experimental data.	Integrates inflammatory and mechanical processes.	Identifiability and patient translation remain unresolved.
Paz et al., 2024 [313]	Neural-network loads driving template FE OA classification.	Retrospective 97-knee OAI analysis.	Tests load and cartilage-detail choices in a cohort.	Classification is not prospective progression or treatment utility.
Ma et al., 2025 [314]	Geometric deep-learning surrogate of FE response under meniscal extrusion.	Agreement with parent FE simulations.	Near-real-time spatial prediction.	Validates surrogate fidelity, not biological or clinical truth.
Hoyer et al., 2025 [315]	qMRI biomarker embedding associated with OA incidence/replacement.	Retrospective imaging cohorts.	Foundation for patient-specific data representation.	Static/foundational; no continuous update or multiscale causal model.
Jayakumar et al., 2025 [316]	PROM/clinical-data benefit–risk decision aid.	Randomised clinical evaluation.	Tests decision quality in patients.	Not a mechanistic or continuously updated knee twin.

**Table 6:** Emerging therapeutic targets linked to knee mechanobiology. No entry should be interpreted as an established disease-modifying knee OA indication.

Target	Proposed Mechanobiological Effect	Strongest Evidence Represented	Current Qualification
TRPV4	Modulate osmotic/mechanical Ca^2+^ signalling and matrix homeostasis.	Explant pharmacology; global and cartilage-specific mouse genetics [261,264,279].	Direction of benefit varies by tissue, age and OA model; systemic effects and dosing window are unresolved.
PIEZO1/2	Reduce high-strain Ca^2+^ overload, apoptosis and inflammatory sensitisation.	In vitro injury; chondrocyte-specific double-knockout mouse PTOA; human tissue association [237,260,267,268,280].	No human interventional efficacy; normal sensory and developmental functions raise safety concerns.
NF-κB/p38	Interrupt convergence of inflammatory and mechanical catabolism.	Extensive cell and animal evidence [226,244,277,280].	Network redundancy and systemic immune toxicity complicate chronic inhibition.
RANKL/denosumab	Modify osteoclast and synovial RANK signalling.	Randomised phase 2a erosive hand OA trial; knee animal models and single-arm human cohort [218,222].	Knee structural disease modification is not established; phenotype, duration and skeletal/immune safety matter.
Sclerostin/SOST	Alter Wnt-linked bone formation and osteochondral crosstalk.	Cartilage expression and conflicting genetic/inhibition studies [144,217].	Not a unidirectional target; cartilage and bone effects may diverge.
YAP/TAZ	Modify stiffness- and cytoskeleton-sensitive transcription.	Causal general cell studies and preclinical cartilage evidence [274,275,276,277,278].	Stage- and tissue-specific effects; broad transcriptional and oncogenic risks require local control.
Senescence/autophagy	Remove pro-inflammatory senescent cells or restore cellular quality control.	Mouse PTOA and ageing studies; cell and tissue evidence [191,192,193,194,195,196,197,198,199,200,201,202,203,204,205,206,207,208].	Human efficacy, target-cell specificity and long-term safety remain uncertain.

## Data Availability

No new data were created or analyzed in this study. Data sharing is not applicable to this article.

## Abbreviations

ACL, anterior cruciate ligament; ADAMTS, a disintegrin and metalloproteinase with thrombospondin motifs; AFM, atomic force microscopy; BW, body weight; CaMK, calcium/calmodulin-dependent protein kinase; DAMP, damage-associated molecular pattern; ECM, extracellular matrix; EMG, electromyography; FE, finite element; FCD, fixed charge density; FRPE/FRPVE, fibril-reinforced poroelastic/poroviscoelastic; GAG, glycosaminoglycan; GBD, Global Burden of Disease; GNN, graph neural network; HA, hyaluronic acid; HGO, Holzapfel–Gasser–Ogden; HIF, hypoxia-inducible factor; IL, interleukin; JCF, joint contact force; KL, Kellgren–Lawrence; LINC, linker of nucleoskeleton and cytoskeleton; MCL, medial collateral ligament; ML, machine learning; MMP, matrix metalloproteinase; MRI, magnetic resonance imaging; NFAT, nuclear factor of activated T cells; NF-κB, nuclear factor kappa-B; OA, osteoarthritis; OPG, osteoprotegerin; PCM, pericellular matrix; PCL, posterior cruciate ligament; PINN, physics-informed neural network; PRG4, proteoglycan 4 (lubricin); PROM, patient-reported outcome measure; PTOA, post-traumatic osteoarthritis; qMRI, quantitative magnetic resonance imaging; QLV, quasi-linear viscoelasticity; RANKL, receptor activator of nuclear factor kappa-B ligand; ROS, reactive oxygen species; SASP, senescence-associated secretory phenotype; SED, strain-energy density; SOST, sclerostin; TGF-β, transforming growth factor beta; TIMP, tissue inhibitor of metalloproteinases; TKR, total knee replacement; TNF, tumour necrosis factor; TRPV4, transient receptor potential vanilloid 4; YAP/TAZ, Yes-associated protein/transcriptional coactivator with PDZ-binding motif.

## Appendix A. Mathematical Formulations and Validity Domains

The main text retains only the three relations needed to follow information across scales. The more detailed or textbook-level formulations below are included for reference. They are schematic: implementation requires the boundary conditions, parameter definitions and numerical details in the cited primary sources.

### Appendix A.1. Joint Kinematics and Dynamics

(A1) *e*_2_ = (*e*_3_ × *e*_1_)/‖*e*_3_ × *e*_1_‖

The floating axis e_2_ is the common perpendicular to the femoral flexion axis e_1_ and tibial axial-rotation axis e_3_ [23,24,25]. The construction is valid when the axes are not parallel; landmark definition and extreme flexion remain sources of error.

(A2) *ΣF* = *ma_cm, ΣM_cm* = *Iα* + *ω* × (*Iω*)

The Newton–Euler balances assume each segment is represented as a rigid body with known mass and inertia [26,27]. They return net loads, not the individual muscle or contact contributions.

(A3) *d/dt*(*∂L*/*∂q̇ᵢ*) − *∂L*/*∂qᵢ* = *Qᵢ*

The Lagrange form uses generalised coordinates qᵢ, Lagrangian L = T − V and generalised forces Qᵢ. Constraint choice and model reduction determine its scale of validity.

### Appendix A.2. Muscle and Contact Models

(A4) *min J* = *Σᵢaᵢⁿ*
*subject to ΣᵢrᵢFᵢ *= *Mnet,* 0 ≤ *aᵢ* ≤ 1

Static optimisation resolves muscle redundancy by an assumed cost, often n = 2 or 3 [48,50]. The solution is model dependent and can under-represent co-contraction unless constrained by EMG.

(A5) *F* = *Fmax[a(t)fL*(*l̃*)*fV*(*ṽ*) + *fPE*(*l̃*)]*cosφ*

A Hill-type actuator combines activation a, normalised force–length f L and force–velocity f V relations, passive force f PE and pennation φ [31,51,52]. It is an organ-scale actuator model rather than a molecular description of cross-bridge kinetics.

(A6) *a* = (3*FR*/4*E**)^1/3^*, p*_0_
*=* 3*F*/(2*πa*^2^)

Hertz contact assumes smooth elastic bodies, small strains and no fluid flow [66,67]. It is useful as a reference limit but is inadequate for time-dependent cartilage contact.

### Appendix A.3. Mixture, Ligament and Bone Models

(A7) *∇·σˢ* + *π* = 0, *∇·σᶠ* − *π* = 0*, π* = *K*(*vᶠ* − *vˢ*)

Biphasic phase momentum balances neglect inertia and represent solid–fluid drag through K, related to permeability [84,85,86]. Constitutive stress and mass-balance equations are additionally required.

(A8) *σ*(*t*) = *∫*_0_ᵗ *G*(*t* − *τ*)[*∂σe*(*ε*)/*∂ε*](*∂ε*/*∂τ*)*dτ*

QLV assumes separability of nonlinear instantaneous elasticity σe and reduced time dependence G [124]. This assumption can fail when relaxation itself changes with strain.

*W* = *μ*(*Ī*_1_
*−* 3)/2 + *Σᵢ₌*_1_^2^*{k*_1_/(2*k*_2_)}[*exp*(*k*_2_⟨*κ*(*Ī*_1_ *−* 3) + (1 − 3*κ*)(*Ī*_4_*ᵢ* − 1)⟩₊^2^) − 1] (A9)

This corrected HGO-type form includes two dispersed fibre families and a tension-only Macaulay bracket ⟨·⟩₊ [126,127]. μ is matrix shear modulus; k_1_ and k_2_ control fibre stiffness and stiffening; κ describes dispersion. Incompressibility, reference configuration and fibre architecture must be specified.

(A10) *dρ*/*dt* = *B*(*U*/*ρ* − *k*)*, U* = ½*σ*:*ε*

The strain-energy-density adaptation law updates apparent density ρ outside a prescribed lazy zone [142,143]. B and k are phenomenological and should not be interpreted as universal osteocyte thresholds.

### Appendix A.4. Cell Mechanics and Growth

(A11) *E* = (3*Φ*/2*π*) *ΔP*/(*Lp*/*a*)

For elastic micropipette aspiration, E is the inferred modulus, ΔP the suction pressure, Lp the aspirated length, a the pipette radius and Φ a geometry/wall correction [165]. This dimensionally consistent form requires a homogeneous incompressible half-space approximation and small enough deformation for the chosen solution.

(A12) *G*(*t*) = *G∞* + (*G*_0_ − *G∞*)*exp*(−*t*/*τ*)

The standard-linear-solid relaxation law defines instantaneous modulus G_0_, equilibrium modulus G∞ and time constant τ [167,285]. A single τ is often only an effective fit to multi-timescale cell behaviour.

(A13) *Fcone* = (2/*π*)[*E*/(1 − *ν*^2^)]*tan*(*α*)*δ*^2^*; Fsphere* = (4/3)[*E*/*(*1 − *ν*^2^)]√*R δ*^3/2^

Sneddon/Hertz indentation assumes an ideal conical or spherical tip, homogeneous isotropic elasticity and an appropriate half-space [167]. Substrate effects, adhesion and finite thickness must be controlled.

(A14) *F* = *FeFg*

Finite growth decomposes the deformation gradient into growth Fg and elastic accommodation Fe [304]. It is a kinematic framework; a biological production law and compatibility conditions are still required. All evidence and representative values are drawn from the cited literature. Repository: https://github.com/lysyloxidase/KneeMechBridge accessed on 1 June 2026.
